# Functional Assay for Measuring Bacterial Degradation of Gemcitabine Chemotherapy

**DOI:** 10.21769/BioProtoc.4797

**Published:** 2023-09-05

**Authors:** Serkan Sayin, Amir Mitchell

**Affiliations:** Department of Systems Biology, University of Massachusetts Chan Medical School, Worcester, MA, USA

**Keywords:** Drug degradation, Drug breakdown, Drug modification, Gemcitabine, Gemcitabine deamination, Bacterial Biotransformation, Functional assay, Tumor-microbiome, Cytidine deaminase

## Abstract

Drug biotransformation by the host microbiome can impact the therapeutic success of treatment. In the context of cancer, drug degradation can take place within the microenvironment of the targeted tumor by intratumor bacteria. In pancreatic cancer, increased chemo-resistance against the frontline chemotherapy gemcitabine is thought to arise from drug degradation by the tumor microbiome. This bacterial–drug interaction highlights the need for developing rapid assays for monitoring bacterial gemcitabine breakdown. While chemical approaches such as high-performance liquid chromatography are suitable for this task, they require specialized equipment and expertise and are limited in throughput. Functional cell-based assays represent an alternate approach for performing this task. We developed a functional assay to monitor the rate of bacterial gemcitabine breakdown using a highly sensitive bacterial reporter strain. Our method relies on standard laboratory equipment and can be implemented at high throughput to monitor drug breakdown by hundreds of strains simultaneously. This functional assay can be readily adapted to monitor degradation of other drugs.

Key features

Quantification of gemcitabine breakdown by incubating bacteria that degrades the drug and subsequently testing the growth of a reporter strain on filtered supernatant.

Use of an optimized reporter strain that was genetically engineered to be a non-degrader strain and highly sensitive to gemcitabine.

A high-throughput assay performed in microplates that can be adjusted for identifying bacteria with a fast or slow gemcitabine degradation rate.

The assay results can be compared to results from a standard curve with known drug concentrations to quantify degradation rate.

**Graphical overview**


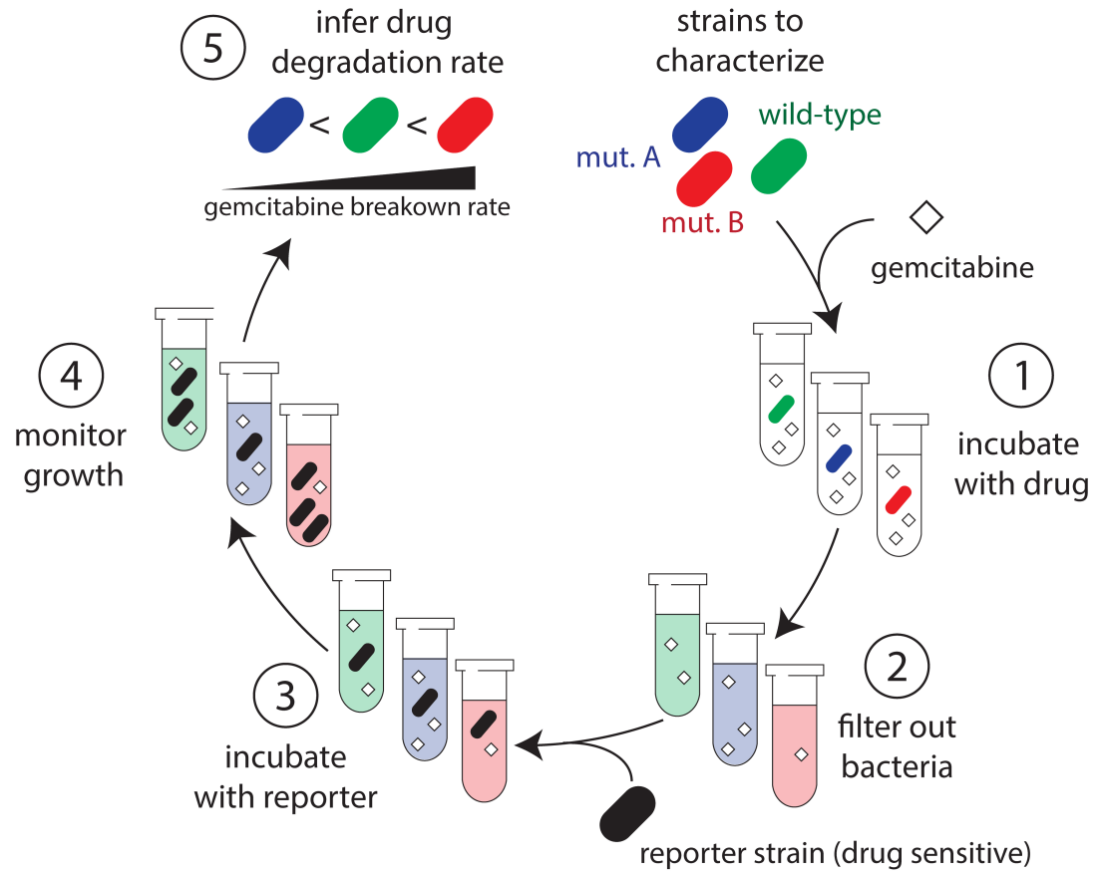


**Protocol overview.** (1) Bacteria are incubated with gemcitabine for a set period of time. (2) Samples are removed from co-incubated suspensions and filtered to remove bacteria to halt gemcitabine degradation. (3) A gemcitabine-sensitive reporter strain is then added to the conditioned supernatant and is supplemented with growth media. (4) Growth of the reporter strain is monitored over time. (5) Results from the growth experiments are used to infer the concentration of gemcitabine in the co-culture supernatant and the drug degradation rate.

## Background

The human microbiome can modulate the efficacy of different therapeutic interventions. In some cases, the effectiveness of host-targeting (non-antibiotic) drugs can be influenced by direct drug–microbiome interactions, such as bacterial drug biotransformation and bioaccumulation (Spanogiannopoulos et al., 2016; Zimmermann et al., 2019 and 2021; Klünemann et al., 2021). In the context of cancer, microbial drug biotransformation can take place in natural microbiome sites, such as the gut, or by tumor-colonizing bacteria. Recent work on pancreatic cancer revealed that gammaproteobacteria, frequently infecting pancreatic tumors in humans, can inactivate the frontline chemotherapy drug gemcitabine [2’,2’-difluoro 2’ deoxycytidine (dFdC)] and increase chemoresistance in murine tumor models (Geller and Straussman, 2017). Published clinical data further provided circumstantial evidence supporting the notion that bacterial drug inactivation may be impacting gemcitabine treatment efficacy in patients (Gao et al., 2020; Meriggi and Zaniboni, 2021; Mohindroo et al., 2021). However, an important and related observation that is often overlooked is that gemcitabine is a potent antimicrobial (Sayin et al., 2023), similar to many other antineoplastic drugs (Maier et al., 2018). We previously proposed and showed that bacteria can rapidly evolve resistance against anticancer chemotherapies that are also antimicrobial, and by doing so can also alter their drug metabolism and therefore inadvertently influence chemoresistance in the treated host (Rosener et al., 2020; Sayin et al., 2023).

Investigating bacterial biotransformation of host-targeted drugs is key for our understanding of cancer–microbiome interactions (Xavier et al., 2020). Chemical methods, such as high-performance liquid chromatography and mass spectrometry, are typically used to quantify drug biodegradation rate by monitoring extracellular drug concentration after bacterial incubation. However, despite their prevalence, these methods require specialized equipment and expertise, and are limited in experimental throughput. In contrast, functional cell-based assays represent an alternative category of approaches for gauging drug concentration by using toxicity as a proxy for its concentration. Specifically, the growth of drug-sensitive cells can be leveraged as a functional reporter for quantifying drug concentration. The use of commonplace microplate optical density (OD) readers for high-throughput monitoring of bacterial growth allows for multiplexing such assays and measuring drug breakdown across hundreds of bacterial cultures with common lab equipment.

We have developed a functional assay to monitor the rate of gemcitabine breakdown in bacteria (Sayin et al., 2023). We used the assay to uncover how loss-of-function mutations in dozens of genes that confer bacterial gemcitabine resistance influence the drug breakdown rate. For this approach, we first incubated the strain of interest in a saline buffer that contained a known drug concentration for set incubation periods. We then filtered the cell suspension and aliquoted the conditioned supernatant into fresh growth media. We inoculated into this media a genetically engineered reporter strain that was optimized for the functional assay—a highly sensitive strain that is incapable of drug degradation. We compared the growth of the reporter strain in multiple supernatants and identified supernatants with altered growth. Increased or decreased growth of the reporter strain was indicative of changes in drug concentration during the incubation period.

## Materials and reagents

**Biological materials**

1. Reporter strain: *Escherichia coli* Δ*cdd* mutant (KEIO knockout collection, Dharmacon, GE Life Sciences)
2. Test strain: any other mutant from KEIO knockout collection or any other bacterial species of interest
3. Reference strain: for KEIO mutants, the parent strain *E. coli* BW25113 (Dharmacon, GE Life Sciences)

**Reagents**

1. Kanamycin monosulfate (TCI, catalog number: K0047)
2. Gemcitabine hydrochloride salt > 99% (LC labs, catalog number: G-4177)
3. Phosphate-buffered saline (PBS) (Corning, catalog number: 21-040-CV)
4. M9 minimal salts (Difco, catalog number: 248510)
5. NaCl (Fisher Scientific, catalog number: S271-3)
6. Yeast extract (Gibco Bacto, catalog number: 212720)
7. Tryptone (Fisher Scientific, catalog number: BP1421-2)
8. MgSO_4_, anhydrous (Sigma-Aldrich, catalog number: M8266-100G)
9. CaCl_2_ (Fisher Scientific, catalog number: C79-500)
10. Protein hydrolysate amicase (Sigma-Aldrich, catalog number: 82514-1KG)
11. Glucose/Dextrose anhydrous (Fisher Scientific, catalog number: D14-500)

**Solutions**

1. Lysogeny broth (LB) (see Recipes)
2. M9 minimal medium (see Recipes)
3. Kanamycin stock solution (50 mg/mL in water) (see Recipes)
4. Gemcitabine stock solution (25 mg/mL in water corresponding to 83.4 mM) (see Recipes)

**Recipes**

1. **Lysogeny broth**
  **Table:** 

Reagent	Final concentration	Quantity
Tryptone	1%	10 g
Yeast extract	0.5%	5 g
NaCl	1%	10 g
Milli-Q H_2_O	n/a	1 L
  Autoclave the solution after dissolving all of the components.
2. **M9 minimal medium**
  **Table:** 

Reagent	Final concentration	Volume
M9 minimal salt solution (5×)	1×	200 mL
20% glucose solution	0.4%	20 mL
1 M MgSO_4_	2 mM	2 mL
1 M CaCl_2_	0.1 mM	0.1 mL
2% protein hydrolysate amicase	0.2%	100 mL
Autoclaved Milli-Q H_ 2_O	n/a	678 mL
  Filter sterilize the components of the M9 minimal medium except the M9 minimal salt solution, by passing through a 0.22 μm syringe filter or 0.22 μm vacuum-driven filter. Autoclave 5× M9 minimal salt solution to sterilize. Heat amicase solution at 42 °C water bath for dissolving. Combine all components in sterile conditions to prepare the final medium.
3. **Kanamycin stock solution**
  Dissolve kanamycin in autoclaved Milli-Q water at 50 mg/mL and filter sterilize by passing through a 0.22 μm PES filter. Keep the stock solution at -20 °C.
4. **Gemcitabine stock solution**
  Dissolve gemcitabine in autoclaved Milli-Q water at 25 mg/mL and filter sterilize by passing through a 0.22 μm PES filter. Keep the stock solution at -20 °C.

**Laboratory supplies**

1. 96-well plate 0.22 μm filters (PALL Corporation, catalog number: 8119)
2. 0.22 μm syringe filter, PES membrane, 30 mm diameter (Celltreat, catalog number: 229747)
3. 10 mL syringes without needle (Air-tite, catalog number: ML10)
4. 0.22 μm vacuum driven filter, PES membrane, 250 mL (GenClone/Genesee, catalog number: 25-225)
5. 96-deep-well plates (Eppendorf, catalog number: 951033502)
6. 96-well plates (Thermo Fisher Scientific, catalog number: 167008)
7. Cuvettes for spectrophotometer (Olympus Plastics, Genesee catalog number: 21-136)
8. Air permeable 96-well plate seals (Excel Scientific, catalog number: BS-25)
9. Adhesive 96-well plate seals (Thermo Scientific catalog number: AB-0558)

## Equipment

1. Plate reader (BioTek Eon/TECAN Spark)
2. Spectrophotometer (Eppendorf, BioPhotometer plus)
3. Microcentrifuge (Eppendorf, Centrifuge 5424)
4. Centrifuge with multi-well plate carriers (Beckman Coulter Avanti centrifuge J-26 XPI with rotor JS-5.3)
5. Shaker incubator (New Brunswick Scientific, Excella E25)
6. Tabletop microplate shaker incubator (Heidolph Inkubator1000)
7. Optional: 96-channel handheld electric pipette (Integra, Viaflo 96)

## Software and datasets

1. MATLAB or any other statistical software (e.g., R, Excel, SPSS)

## Procedure

- **Preparing the conditioned supernatant**
  Figure 1A shows the overview of the procedure. For biological replicates, we recommend repeating the entire section A on different days.
  **Figure 1.**
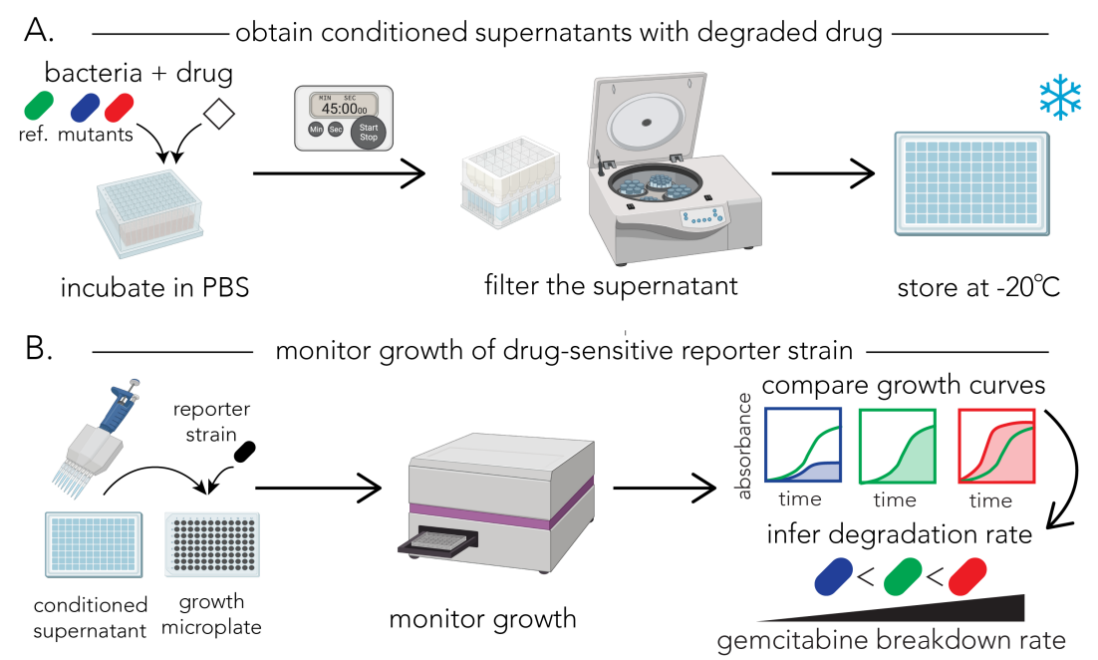
Schematic representation of the protocol for determining gemcitabine degradation rate. A. Protocol for preparing the conditioned supernatants. Bacterial cultures, normalized to an identical optical density, are incubated with gemcitabine in PBS for defined periods of time. Samples are removed from the incubated cultures and filtered to yield conditioned supernatants. The assay can be paused after the filtering stage and supernatants can be frozen for later use. B. Protocol for the functional assay. The reporter strain (*E. coli* Δ*cdd)* is grown overnight and aliquots of the conditioned supernatant are then added to the growth culture. Growth of the reporter strain is monitored in a microplate optical density (OD) reader. Changes in growth curves are used to infer the gemcitabine concentration in the conditioned supernatant of different strains (red: fast degrader; blue: slow degrader) compared to a reference strain (green). Parts of the figure were created with BioRender.com.
  1. Inoculate your test bacterial strains into 1 mL of LB in a 96-deep-well plate and grow overnight at 37 °C, 200 rpm shaker. Also inoculate the reference strain for comparison. **Critical:** Inoculate multiple replicates of reference strain (at least six technical replicates)
  2. On the next day, pellet the cells by centrifuging the plate at 5,000× *g* for 10 min using a centrifuge with multi-well plate holders.
  3. Discard the supernatant without disrupting the pellet and resuspend the cells in 1 mL of PBS.
  4. Pellet the cells by centrifugation at 5,000× *g* for 10 min using a centrifuge with multi-well plate holders.
  5. Repeat steps A3–A4 two additional times.
  6. Let the cells incubate in 1 mL of PBS for 1 h and then measure the OD (600 nm) after 1:4 dilution in PBS at a final volume of 200 μL (e.g., 50 μL of cell suspension, 150 μL of PBS).
  7. Dilute the cultures to an OD of 0.5 in a 96-deep-well plate at a final volume of 1.1 mL of PBS.
  8. Sample 200 μL from the diluted cultures and measure optical density to validate if the dilution was accurate. **Critical:** Accuracy of dilution is important since any deviation from the intended cell number will influence the drug breakdown speed and will be incorrectly interpreted as a change in drug breakdown rate.
  9. Add gemcitabine at a final concentration of 200 μM: prepare 21× (4.2 mM) gemcitabine and pipette 45 μL into 900 μL of bacterial suspension. **Critical:** Make sure your gemcitabine stocks are kept sterile and avoid multiple freeze-thaw cycles.
    **Caution:** Always wear appropriate personal protective equipment when working with gemcitabine and follow institutional health and safety regulations for managing gemcitabine waste. Gemcitabine is a cytotoxic chemotherapy agent.
  10. Incubate the plate on a tabletop microplate shaker at 37 °C and 900 rpm.
  11. At designated timepoints (15 and 45 min), transfer 400 μL of liquid culture into a 96-well plate filter placed onto a 96-deep-well plate and centrifuge immediately at 5,000× *g* for 5 min using a centrifuge with multi-well plate holders. **Critical:** A 15 min timepoint is suitable for identifying fast degraders, and a 45 min timepoint is suitable for identifying the slow degraders. These incubation periods can be adjusted for work with other bacterial species that degrade gemcitabine at considerably different rates than *E. coli* .
  12. Safe stopping point: if you are not proceeding immediately to section B, cover the supernatant with sterile plastic foil and freeze at -20 °C.
- **Performing the functional assay: testing the conditioned supernatants**
  Figure 1B shows the overview of the procedure. We recommend performing section B in triplicates using three independent biological replicates that are collected by performing section A on three different days.
  1. Inoculate a single colony of reporter mutant Δ*cdd* into 3 mL of M9 media with 50 μg/mL kanamycin and grow overnight at 37 °C and 200 rpm in a shaker.
  2. On the next day, measure the OD of the culture (after 1:20 dilution in M9) and dilute the culture to OD 1 in 1 mL of M9.
  3. Further dilute the culture 1:500 in M9 (final volume of 25 mL) and pipette 190 μL into each well of a 96-well microplate.
  4. Add 10 μL of conditioned buffer from section A into each well. This step should be performed quickly, so all individual cultures start the exposure at the same time point. **Caution:** If the conditioned supernatants to be tested are frozen, thaw them at room temperature in advance.
    **Optional:** This step can be performed using a 96-channel handheld electric pipette to make sure all wells receive the conditioned supernatant simultaneously.
  5. Monitor the growth of the culture in the microplate using a suitable microplate optical density reader. Define a repeating measurement cycle that includes incubation at 37 °C and double orbital shaking at 360 rpm with reading the OD every 10 min over 8 h (plate lid on). Make sure to measure M9 alone for a blank optical density value.

## Data analysis

**Identifying the fast and slow degraders**

1. Subtract the blank optical density value from all data points. Alternatively, you can treat the average optical density at the first timepoint across all wells as the background optical density.
2. Plot the growth curves for each strain as time on *x*-axis and OD on *y*-axis for each replicate.
3. Calculate the area under the curve (AUC) up to 7 h of growth for each replicate.
4. Perform one-tailed paired Student’s *t*-test with test strain AUCs and reference strain AUCs.
  Important: pick the correct tail of the distribution, depending on your interest in slow or fast degraders. For example, for determining fast degraders, using 15 min conditioned supernatant, use a right tail.
5. Adjust the *p*-values that were calculated with a false discovery rate (FDR) or Bonferroni correction. Determine the hits based on your p-adjusted cutoff (generally < 0.1 or < 0.05).

## Validation of protocol

**Figure 2.**
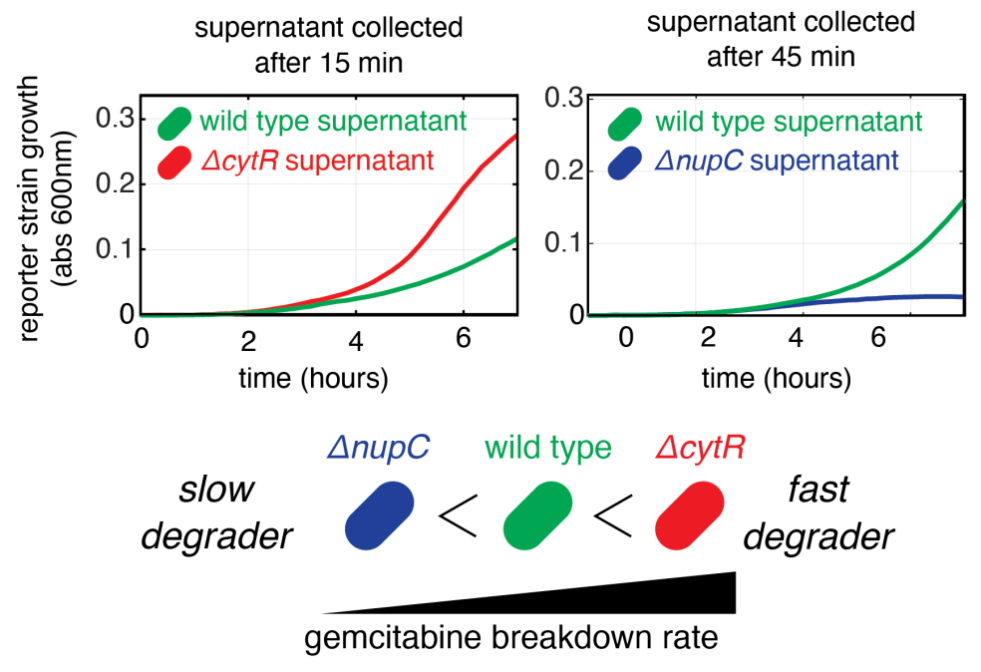
Representative results from the assay. Graphs show the growth curves of the reporter strain on conditioned supernatants after incubation with three *E. coli* strains: wild type, *ΔcytR* (fast degrader), and *ΔnupC* (slow degrader). The graph shows the mean of three replicates.

This protocol was used to produce data on panel B of Figure 2 in our previous publication (Sayin et al., 2023). Section A has been repeated as three biological replicates and section B has been performed three times with the supernatants collected from biological replicates performed on different days. For the reference strain, six technical replicates have been used with the wild-type *E. coli* strain. Other controls used for validation were a no-bacteria control and no-gemcitabine control. Since multiple hypothesis testing was performed, *p-*values calculated using one tailed Student’s *t*-test were adjusted using FDR and a cutoff of < 0.1 was used. Measurement of drug degradation from our functional assay for a fast degrader and a slow degrader were validated with a chemical method (gas chromatography–mass spectrometry) that measured drug concentration and its degradation product. Figure 2 shows examples of fast and slow gemcitabine degraders.

## General notes and troubleshooting

**General notes**

We expect that the rate of gemcitabine degradation may differ considerably between different bacterial species. The functional assay can be adjusted to account for such differences. The following parameters may need to be optimized for work with other species:

1. **Bacterial concentration:** bacterial concentration in step 7 of section A should be lowered for work with fast degraders.
2. **Incubation period:** incubation period in step 11 of section A should be shortened for working with fast degraders.
3. **Initial gemcitabine concentration:** drug concentration in step 9 of section A can be decreased for slow degraders.

Multiple controls in section A must be included for sensible interpretation of the experiment results:

1. **No-bacteria, no-gemcitabine control:** for observing unhindered growth of the reporter strain.
2. **No-bacteria, +gemcitabine:** for validating that maximum gemcitabine concentration is indeed inhibitory for the reporter strain.
3. **+bacteria, no-gemcitabine:** for validating that bacteria are not secreting molecules into the supernatant that inhibit growth of the reporter strain.
4. **+reference bacteria, +gemcitabine:** for determining the gemcitabine breakdown in a reference strain (e.g., wild-type strain).

**Limitations of the protocol**

Technical artifacts in the functional assay can be incorrectly interpreted as strains with fast or slow degradation. Great attention should be therefore dedicated to validating that cell numbers and incubation periods are identical across all cultures. Moreover, microplate optical density readers can typically have slightly uneven growth conditions across the multi-well plates (e.g., non-uniform heating). To minimize these artifacts, we recommend shuffling the positions of biological replicates across the plate.

**Applicability to other experimental systems/model organisms**

This protocol can be used with any bacteria that can be cultured in the lab. This experimental approach can also be used to measure the degradation of other drugs, as long as a sensitive reporter strain can be identified.

## References

[1] Gao Y., Shang Q., Li W., Guo W., Stojadinovic A., Mannion C., Man Y. g. and Chen T. (2020). Antibiotics for cancer treatment: A double-edged sword. J. Cancer 11 (17): 5135-5149. 32742461 10.7150/jca.47470 PMC7378927

[2] Geller L. T. and Straussman R. (2017). Intratumoral bacteria may elicit chemoresistance by metabolizing anticancer agents. Mol. Cell. Oncol. 5(1): e1405139. 29404397 10.1080/23723556.2017.1405139 PMC5791857

[3] Klünemann M., Andrejev S., Blasche S., Mateus A., Phapale P., Devendran S., Vappiani J., Simon B., Scott T. A., Kafkia E., .(2021). Bioaccumulation of therapeutic drugs by human gut bacteria. Nature 597( 7877): 533-538. 34497420 10.1038/s41586-021-03891-8 PMC7614428

[4] Maier L., Pruteanu M., Kuhn M., Zeller G., Telzerow A., Anderson E. E., Brochado A. R., Fernandez K. C., Dose H., Mori H., .(2018). Extensive impact of non-antibiotic drugs on human gut bacteria. Nature 555( 7698): 623-628. 29555994 10.1038/nature25979 PMC6108420

[5] Meriggi F. and Zaniboni A. (2021). Antibiotics and steroids, the double enemies of anticancer immunotherapy: a review of the literature. Cancer Immunol., Immunother. 70(6): 1511-1517. 33165628 10.1007/s00262-020-02786-3 PMC10991597

[6] Mohindroo C., Hasanov M., Rogers J. E., Dong W., Prakash L. R., Baydogan S., Mizrahi J. D., Overman M. J., Varadhachary G. R., Wolff R. A., .(2021). Antibiotic use influences outcomes in advanced pancreatic adenocarcinoma patients. Cancer Medicine 10(15): 5041- 5050. 34250759 10.1002/cam4.3870 PMC8335807

[7] Rosener B., Sayin S., Oluoch P. O., García González A. P., Mori H., Walhout A. J. and Mitchell A. (2020). Evolved bacterial resistance against fluoropyrimidines can lower chemotherapy impact in the Caenorhabditis elegans host. eLife 9: e59831. 33252330 10.7554/eLife.59831 PMC7725501

[8] Sayin S., Rosener B., Li C. G., Ho B., Ponomarova O., Ward D. V., Walhout A. J. and Mitchell A. (2023). Evolved bacterial resistance to the chemotherapy gemcitabine modulates its efficacy in co-cultured cancer cells . eLife 12: e83140 . 36734518 10.7554/eLife.83140 PMC9931390

[9] Spanogiannopoulos P., Bess E. N., Carmody R. N. and Turnbaugh P. J. (2016). The microbial pharmacists within us: a metagenomic view of xenobiotic metabolism. Nat. Rev. Microbiol. 14(5): 273- 287. 26972811 10.1038/nrmicro.2016.17 PMC5243131

[10] Xavier J. B., Young V. B., Skufca J., Ginty F., Testerman T., Pearson A. T., Macklin P., Mitchell A., Shmulevich I., Xie L., .(2020). The Cancer Microbiome: Distinguishing Direct and Indirect Effects Requires a Systemic View. Trends Cancer 6(3): 192- 204. 32101723 10.1016/j.trecan.2020.01.004 PMC7098063

[11] Zimmermann M., Patil K. R., Typas A. and Maier L. (2021). Towards a mechanistic understanding of reciprocal drug–microbiome interactions. Mol. Syst. Biol. 17(3): e202010116. 10.15252/msb.202010116 PMC7970330 33734582

[12] Zimmermann M., Zimmermann-Kogadeeva M., Wegmann R. and Goodman A. L. (2019). Mapping human microbiome drug metabolism by gut bacteria and their genes. Nature 570 (7762): 462-467. 31158845 10.1038/s41586-019-1291-3 PMC6597290

